# A cross-sectional study on patient-centered care in a selected hospital in eThekwini district, KwaZulu-Natal, South Africa

**DOI:** 10.4102/hsag.v30i0.2913

**Published:** 2025-03-31

**Authors:** Marcel Peruma, Waheedha Emmamally, Mildred Mooi, Uchenna B. Okafor

**Affiliations:** 1 Department of Nursing Science, College of Science, University of KwaZulu-Natal, Westville, South Africa; 2 Department of Nursing Science, Faculty of Health Sciences, Walter Sisulu University, Mthatha, South Africa

**Keywords:** patient-centred care provision, critical nurses, patients, high-quality healthcare, South Africa

## Abstract

**Background:**

Clinical healthcare reform demands high-quality patient care, especially in emergencies. Patient-centred care (PCC) prioritises therapy based on health, characteristics, and needs.

**Aim:**

This study examines critical care nurses’ views on PCC in a hospital in eThekwini, KwaZulu-Natal.

**Setting:**

The study was conducted at a selected tertiary care facility in eThekwini district, KwaZulu-Natal, South Africa.

**Methods:**

This cross-sectional study examined 119 conveniently selected critical care nurses from five units treating critically ill adult patients in a central tertiary care hospital in eThekwini district, KwaZulu-Natal, South Africa. Personified patient care was measured using the Individualised Care Scale (ICS).

**Results:**

The mean values for nurse-supported customised care ranged from 4.27 ± 0.66 to 4.44 ± 0.61. Fear and anxiety during patient discussions have the highest mean score (4.44 ± 0.61). The mean values for four personal life statements were 4.22 ± 0.72 to 4.29 ± 0.65. Hospitalisation experience was surveyed by 90.8% of people, with a mean score of 4.29 ± 0.61. Patients’ desire to understand their illness was surveyed by nurses (91.60%) with a mean score of 4.39 ± 6.39. The majority (94.9%) of nurses encouraged patients to express care preferences, whereas 85.8% were inquired about their preferred bathing time. The majority (59.70%) scored average, while 38.70% high.

**Conclusion:**

Patient-centred care support was average among critical care nurses. Training and education in critical care should emphasise PCC. To strengthen PCC in clinical practice, execute PCC activities regularly.

**Contribution:**

The study revealed PCC actions and indicated critical care nurses’ average support.

## Introduction

The need to provide optimal quality healthcare for patients, particularly in critical health conditions, is a fundamental tenet in strengthening clinical healthcare and putting patients at the core of healthcare. Thus, the conceptualisation and evolution of patient-centred care (PCC) concerns the desire to centralise or prioritise the care of patients, given their unique health situation, traits and needs. Patient-centred care means providing personalised or individualised care to patients in a clinical health setting. It is an act of offering care to patients in a friendly, humanising, emphatic and loving manner, thereby enabling the patient take ownership of the health and lives. According to Kumar and Chattu (2018), PCC entails shifting the focus of care from patients to persons, enhancing the person’s individuality and uniqueness, and in the process, this type of care creates a partnership among healthcare professionals, patients and patient families to ensure that care delivered is attentive to the needs, values and preferences of patients (Dong, Jameel & Gagliardi 2022).

Critical care nurses place critically ill or unconscious patients in a critical care health facility. Often, family members are desirous and anxious about their patients’ health condition and what form of assistance or care they could provide. In this context, a nurse–patient–family relationship becomes inevitable. Thus, family members form a valuable resource for contemporary healthcare delivery in intensive care units (Goldfarb et al. 2022). Families can actively participate in communication and decision-making with the healthcare team, offer emotional or physical support to their loved ones, and fully contribute to the delivery of care (Hamilton et al. 2020). Patient-centred care include critically ill patients, their families and critical care nurses.

Creating initiatives to promote PCC has many benefits for the patient and family members. Positive healthcare outcomes include increased patient and family satisfaction with care, shorter hospital stays, low healthcare costs, greater autonomy and greater patient involvement in their care (Rosengren et al. 2018). Empirical research evidence has shown that patients value healthcare professionals spending time with them, explaining procedures, and involving them in decisions regarding their health as opposed to the technical aspects of care (Kwame & Petrucka 2021; McAdam et al. 2012; Van Mol et al. 2017). Despite the numerous benefits of PCC in improving patient health outcomes, nurses who are tasked with providing or implementing PCC to patients in critical health conditions often fail to perform this caring act because of various factors such as ignorance, health system-related challenges such as staff shortages, busy schedules, technological barriers and sheer negligence. Furthermore, environmental barriers to PCC provision include patient isolation from family members in a noisy and complicated environment (Kang et al. 2019). These patients are often overwhelmed with anxiety and fear. Therefore, the negative professional practice may act as a barrier to PCC. The professional practice environment should ideally support nurses to work effectively in a multidisciplinary team, to function at the highest scope of nursing practice, and to mobilise resources quickly (Zeleníková et al. 2020). The characteristics of the critical care environment impact the application of a PCC approach to care, and a positive work environment is associated with a significantly higher quality of care and vice versa (Kieft et al. 2014).

In the South African context, the critical care environment constitutes a barrier to the provision of PCC by South African nurses because of a lack of resources (Joynt et al. 2019). South Africa has a resource-limited public health system that caters to 84% of the entire population and accounts for 43% of the country’s intensive care unit (ICU) beds, with 8.9 ICU beds per 100 000 people, unlike the United States, which has more than 30 ICU beds per 100 000 people (Anesi et al. 2020). South Africa’s unique disease burden further complicates the situation, impacting the professional practice environment. However, the expectation for nurses to perform their duties in severely under-resourced environments and meet the needs of a diverse and unique population makes PCC provision more challenging. The coronavirus disease 2019 (COVID-19) pandemic complicated this scenario by causing millions of people to become critically ill and require critical care units (CCU) care (Simpson & Robinson 2020); however, critical care nurses, as frontline workers, faced limited resources and a lack of personal protective equipment (Rangachari & Woods 2020). The obstacles present in the South African healthcare system hinder the delivery of PCC, leading to the perception that critical care nurses fail to apply their theoretical knowledge to the principles of critical care practice (Baboo, Van Rooyen & Ricks 2016). It is important to know how critical care nurses feel about PCC activities for critically ill patients with rare medical conditions that need very specialised care. This is because it helps us understand what the Batho Pele Principles and the Patients’ Rights Charter are about and how they should be applied to healthcare in South Africa (Pretorius & Klopper 2012). Therefore, the authors designed this study to evaluate the critical care nurses’ perception of PCC activities at a selected hospital in eThekwini district, KwaZulu-Natal, South Africa.

## Research methods and design

### Research setting

This cross-sectional study included 119 conveniently selected critical nurses who worked in five designated CCUs for critically ill adult patients at a selected hospital in a central and tertiary care facility in eThekwini District, KwaZulu-Natal, South Africa. The hospital has a total of 846 beds catering to the needs of the population. As a central hospital, it provides highly specialised care to the population of KwaZulu-Natal. The hospital has paediatric, neonatal and adult CCUs.

### Sampling and sample size

Participants were included in the study if nurses registered with the South African Nursing Council, either with a Diploma in Nursing or a Degree in Nursing, and critical care nurses with or without an additional qualification in critical care nursing, working in the CCU at the time of data collection and willing to participate in the study. However, the study excludes enrolled nurses (2-year certificate course in Nursing) working in the CCUs.

To estimate the proportion of critical care nurses with an adequate perception of PCC, a sample size of 100 was necessary, assuming a 95% probability and a baseline percentage of 50%. Anticipating a 20% non-response rate, the required sample size was increased to 130 nurses. From the sampling frame of approximately 77% nurses, the authors randomly selected 119 for the survey.

### Data collection instrument

The authors collected data on the demographic variables (gender, age, highest nursing qualification, and number of years of experience in the present CCU) using a questionnaire. The authors also utilised the Individualised Care Scale (ICS) measuring tool, developed by Suhonen, Gustafsson and Katajisto (2010), to gather pertinent data on various aspects of individualised patient care. The ICS has 17 items on a 5-point Likert scale (1 = strongly disagree, 2 = disagree, 3 = neither agree nor disagree, 4 = agree, 5 = strongly agree), which solicit the nurse’s opinion about individualised care based on their daily nursing activities. The ICS-Nurse consists of three subscales. The first subscale has seven items related to the clinical situation, including physical and psychological care needs, fears and anxieties, abilities or capacities, health condition, meaning of illness, reactions or responses to illness, and feelings or affective states.

The second subscale has four items measuring personal life situation, including life situation in general and daily activities, habits or preferences, cultural background or traditions, family involvement and earlier experiences of hospitalisation. The third subscale consists of six items that pertain to decisional control over care. These items include knowledge about illness and treatment, the ability to make choices and consider alternatives, decision-making skills, and the ability to express personal views, opinions, wishes, or make proposals. The minimum score for ICS-A-Nurse is 17, and the maximum is 85. Higher scores of the ICS indicate more nurse-supported individualised patient care, while lower scores signify low individualised patient care support. The ICS-A internal consistency reliability scale had a strong alpha reliability score of 0.94 (Suhonen, Leino-Kilpi & Välimäki 2005). This study achieved the internal consistency of the questionnaire through a pilot study of five critical care nurses, which was not part of the actual data analysis.

### Data collection procedure

Prior meetings with the nursing management were conducted to negotiate for the recruitment of participants and for subsequent data collection. A scheduled time was arranged with the critical care nurses willing to participate in the study. After signing informed consent, the questionnaire was administered to the willing participants in a sealed envelope. The questionnaire was completed on-site and collected bi-weekly.

### Data analysis

The authors summarised the participants’ perceptions of PCC for ICS-A across all three subscales using descriptive statistics such as frequency counts, percentage distribution, mean and standard deviation. The authors applied the Chi-square test to assess the relationship between the participants’ sociodemographic variables and the clinical, personal life situation, and decisional control in individualised care provision (Table 4). The significance level was set at *p* < 0.05.

### Ethical considerations

The study protocol was approved by the University of KwaZulu-Natal Ethics Committee (Ref. no.: BREC/00002791/2021). Permission to conduct the study was obtained from the Department of Health, the Nursing Service Manager, and the operational managers of the critical care units at the selected health facilities. Written informed consent was obtained from participants prior to the data collection. The purpose of the study was explained to the participants, as well as the voluntary nature and right to withdraw at any time. The participants’ identity was anonymous using coding and the confidentiality of their information concealed. The study was conducted according to the principles of the Helsinki Declaration (2013).

## Results

Out of the 150 questionnaires distributed, 119 were retrieved and correctly filled, indicating a response rate of 79%. Most of the participants (84.90%) were females, between 31 and 49 years (73.10%; *n* = 87), had 6–10 years of experience (39.50%; *n* = 47) and had a diploma in nursing (48.70%; *n* = 58).

### Clinical situation

The mean score of 4.44 ± 0.61 relates to fears and anxieties while talking to patients. Most of the participants (95.80%, *n* = 114) expressed the feeling of fear and anxiety while talking to patients. Similarly, 91.60% (*n* = 109) of nurses give patients the opportunity to take responsibility for their care. Furthermore, Table 1 shows that 88.30% (*n* = 105) of nurses asked patients about the impact of their illness or health condition.

**TABLE 1:** Clinical, personal life situational and decisional control nursing support individualised care activities.

Statements	Strongly disagree		Disagree		Neutral		Agree		Strongly agree		Mean ± s.d.
	*n*	%	*n*	%	*n*	%	*n*	%	*n*	%	
**Clinical situation**											
I talk with patients about the feelings they have about their illness/health condition	0	0.00	1	0.80	1	0.80	69	58.00	48	40.30	4.38 ± 0.55
I talk with patients about their needs that require care and attention	0	0.00	1	0.80	4	3.40	59	49.60	55	46.20	4.41 ± 0.60
I gave patients the chance to take responsibility for their care as far as they are able	0	0.00	1	0.80	9	7.60	51	42.90	58	48.70	4.39 ± 0.66
I identify changes in how they have felt	0	0.00	2	1.70	8	6.70	58	48.70	51	42.90	4.33 ± 0.67
I talk with patients about their fears and anxieties	0	0.00	1	0.80	4	3.40	56	47.10	58	48.70	4.44 ± 0.60
I try to find out how their illness or health condition has affected them	0	0.00	0	0.00	14	11.80	59	49.60	46	38.70	4.27 ± 0.88
I talk with patients about what the illness or health condition means to them	0	0.00	1	0.80	10	8.40	58	48.70	50	42.00	4.32 ± 0.66
**Personal life situation**											
I ask patients what kinds of things they do in their everyday life outside the hospital (work, leisure activities)	0	0.00	0	0.00	15	12.60	53	44.50	49	41.20	4.25 ± 0.73
I ask patients about their previous experience of hospitalisation	0	0.00	1	0.80	10	8.40	62	52.10	46	38.70	4.29 ± 0.65
I ask patients about their everyday habits (e.g. personal hygiene)	0	0.00	2	1.70	15	12.60	57	47.90	45	37.80	4.22 ± 0.72
I ask patients whether they want their family to take part in their care	0	0.00	3	2.50	16	13.40	52	43.70	48	40.30	4.22 ± 0.77
**Directional situation**											
I give instructions to patients using language that is easy to understand	2	1.70	1	0.80	7	5.90	40	33.60	69	58.00	4.45 ± 0.78
I ask patients what they want to know about their illness or health condition	0	0.00	0	0.00	10	8.40	53	44.50	56	47.10	4.39 ± 0.63
I listen to patients’ personal wishes about their care	0	0.00	2	1.70	7	5.90	57	47.90	53	44.50	4.35 ± 0.67
I help patients take part in decisions concerning their care	0	0.00	1	0.80	5	4.20	61	51.30	52	43.70	4.38 ± 0.61
I encourage patients to express their opinions on their care	0	0.00	1	0.80	5	4.20	68	57.10	45	37.80	4.32 ± 0.59
I ask patients at what time they would prefer to wash	1	0.80	5	4.20	11	9.20	56	47.10	46	38.70	4.18 ± 0.83

s.d., standard deviation.

### Personal life situation

The mean values for the four personal life situation statements ranged from 4.22 ± 0.72 to 4.29 ± 0.65 (Table 1). Most of the participants (90.8%, *n* = 108) asked patients about their previous hospitalisation experience, with a mean score of 4.29 ± 0.61. Similarly, 85.7% (*n* = 102) of the nurses asked patients what kinds of things they do in their everyday life outside of the hospital, including work or leisure activities, with a mean score of 4.25 ± 0.73. In addition, 85.7% (*n* = 102) of the nurses asked patients about their everyday habits, such as personal hygiene. Most of the respondents (84.0%, *n* = 100) confirmed that they asked the patients if they would like their family to be involved in their care.

### Directional control

The mean values for the six decisional control subscales ranged from 4.18 ± 0.83 to 4.45 ± 0.78. Most participants (91.60%, *n* = 109) indicated providing instructions to patients using a language that is easy to understand, with a mean score of 4.45 ± 0.78. About 91.60% (*n* = 109) of the nurses enquired about patients’ desire to understand the nature of their illness or health condition, with a mean score of 4.39 ± 6.39. About 94.9% (*n* = 113) of the nurses encouraged patients to express their opinions on their care. About 85.8% (*n* = 102) of the nurses inquired about the patient’s preference for wash times (Table 1).

The minimum score for the respondents was 47 and the maximum score was 85. The mean score for the ICS-A-Nurse was 73.60 ± 7.50. The scores with < 50 were regarded as poor support for PCC, between 50 and 75 as average support and 76–85 as high support. Most of the respondents (59.70%, *n* = 71) had an average score, while 38.70% (*n* = 46) had a high support score (Table 2).

**TABLE 2:** Measuring Individualised Care Scale A-Nurse scores.

Variables	*p*		Av		H		Total		Chi-square value	*df*	*p*
	*n*	%	*n*	%	*n*	%	*n*	%			
**Age (years)**											
< 30	8	6.70	5	7.00	3	6.30	8	6.70	1.16	2	0.55
31–49	87	73.10	54	76.10	33	68.80	87	73.10	-	-	-
> 50	24	20.20	12	16.90	12	25.00	24	20.20	-	-	-
**Gender**											
Male	101	84.87	12	16.90	5	10.40	17	14.30	2.38	1	0.30
Female	18	15.13	59	83.10	42	87.50	101	84.90	-	-	-
**Years of experience**											
< 1	1	0.80	0	0.00	1	2.10	1	0.80	4.91	3	0.17
1–5	32	26.90	20	28.20	12	25.00	32	26.90	-	-	-
6–10	47	39.50	32	45.10	15	31.30	47	39.50	-	-	-
> 10	39	32.80	19	26.80	20	41.70	39	32.80	-	-	-
**Highest qualification in nursing**											
Critical care nursing	26	21.80	13	18.30	13	27.10	26	21.80	1.88	3	0.59
Diploma	58	48.70	36	50.70	22	45.80	58	48.70	-	-	-
Bachelor’s degree	34	28.60	21	29.60	13	27.10	34	28.60	-	-	-
Master’s degree	1	0.80	1	1.40	0	0.00	1	0.80	-	-	-

Note: The Pearson Chi-square test showed no significant association between the demographic variables and the overall scores of the ICS-A-Nurse scores.

*df*, degrees of freedom; AV, average; H, high.

## Discussion

Providing PCC is a multidimensional task involving governmental health system support and nursing commitment and willingness. The study’s findings revealed that most nurses expressed a feeling of fear and anxiety when talking to patients, providing patients with the opportunity to take responsibility for their care, and asking patients about the impact of their illness or health condition. This observation aligns with Galehdar et al. (2020), who express concern about nurses experiencing various forms of psychological and mental distress.

Regarding personal circumstances, most participants inquired about patients’ prior hospital experiences, their daily activities outside the medical facility, including professional and recreational pursuits, and their routine habits, such as personal care routines. Furthermore, the nurses asked the patients if they would like their family to be involved in their care. The directional control domains reveal that most participating nurses provide instructions to patients in an easy-to-understand language, inquire about the patients’ desire to understand the nature of their illness or health condition, encourage patients to express their opinions on their care, and consider the patient’s preference for washing times. Impressively, most of the nurses (59.70%) had average support for PCC, while 38.70% had high support. However, there was no significant association between the demographic variables and the overall PCC scores.

**TABLE 3:** Classification of patient-centred Care Scale.

PCC rating	Score	*n*	%
Poor support of PCC	Below 50	2	1.70
Average support of PCC	50–75	71	59.70
High support of PCC	76–85	46	38.70

**Total**	**85**	**119**	**100.00**

PCC, patient-centred care; AV, average; H, high; P, poor.

**TABLE 4:** Sociodemographic characteristics of participants (*N* = 119).

Variables	*n*	%
**Age (years)**		
< 30	8	6.70
31–49	87	73.10
> 50	24	20.20
**Gender**		
Male	101	84.87
Female	18	15.13
**Years of experience**		
Less than 1	1	0.80
1–5	32	26.90
6–10	47	39.50
> 10	39	32.80
**Highest nursing qualification**		
Additional qualification in critical care nursing	26	21.80
Diploma	58	48.70
Bachelor’s degree	34	28.60
Master’s degree	1	0.80

Note: The Table displays the clinical, personal, situational and decisional control nursing support individualised care activities. The mean values for the nurse-supported individualised care activities ranged from 4.27 ± 0.66 to 4.44 ± 0.61.

Nursing care for critically ill patients should address their emotional and spiritual concerns, including anxiety because of uncertainty, fear, financial impact or family effect (Albaqawi, Butcon & Molina 2017; Ahrens 2021). It was encouraging to notice that the nursing activity with the highest mean support score (4.44) in Subscale 1 (Clinical Situation) related to nurses talking to patients about their fears and anxieties. The authors can link this nursing activity to one of the dimensions of PCC, which involves providing emotional support to patients.

Yoo, Lim and Shim (2020) found, contrary to this study’s results that a critical care nurse’s communication with patients primarily focused on biomedical issues rather than addressing patients’ fears or anxieties. The authors found that nurses often communicated with patients regarding vital signs, pain management and hygiene (Slatore et al. 2014). Ahmad (2005) conducted a study on communication with critically ill patients and found that critical care nurses generally viewed communication as a crucial aspect of good nursing. However, (Istanboulian et al. 2022) stated that evidence suggests that communication in the critical care unit was not effectively and consistently applied and seen as a barrier to getting work done.

The nursing activity with the lowest mean score (4.20) involved a nurse asking patients about the impact of their illness or health condition. Yoo et al. (2020) conducted a study that revealed critical care nurses faced more challenges in communicating with patients than in performing essential nursing activities. The communication problems experienced by the nurses were predominantly between nurses and patients. The urgency of care required for critically ill nursing patients is related and attributed to this phenomenon. Happ et al. (2011) discovered that nurses often initiated communication exchanges with their patients, focussing on nurse assessment, care provision and patient needs, rather than the impact of the patient’s illness or health condition.

The nursing activity with the highest mean support score, relating to personal life situations, demonstrated critical nurses asking patients about their previous experiences of hospitalisation (mean = 4.44). Karlsson, Forsberg and Bergbom (2012) emphasised that for nurses to communicate caringly to their patients, they need to set aside time to build a trusting relationship. Questions about previous hospital stays can help build a trusting relationship and will seek to educate critical care nurses about the patient’s preferences. The results from this study are consistent with the results of Al-Shamaly (2022), revealing that nurses rated the importance of being there for their patients and building trusting relationships as integral to providing high-quality nursing care.

Family involvement in patient care is one of the six dimensions of PCC and is critical to its provision. According to Olding et al. (2015), patients admitted to hospitals are members of a wider patient–family network that functions as a small social system. Acknowledging family members in this form leads one away from the disease-centred practice of focussing on the physical care of an individual patient within the intensive care unit. It was disconcerting that the nursing activity relating to the nurse asking patients whether they want their family to participate in their care had the lowest mean support score (4.22) in Subscale 2. McConnell and Moroney (2015) found that critical care nurses adopt a paternalistic approach when considering family involvement in patient care. The critical care patient, the critical care relative, the critical care nurse and the critical care environment are identified as contributing to the difficulties surrounding family involvement in patient care (McConnell & Moroney 2015).

In terms of decisional control, the nurses in this study showed strong support (mean = 4.45) for providing instructions to patients using a language that is easy to understand. A previous study by Işık and Yıldırım (2021) also found that critical care nurses were keen to communicate with patients in a language that was easy for the patients to understand. The ability to communicate can be one of the greatest assets or greatest liabilities of a critical care nurse (Brindley et al. 2014). Critical care nurses must prioritise building relationships with patients by communicating in a language that the patient perceives as patient-friendly.

The respondents in the study showed the least support for the nursing activity of asking patients their preference on wash times (mean = 4.18). Coyer, O’Sullivan and Cadman (2011) also reported this finding, stating that nurses failed to communicate with patients about hygiene and did not provide flexible times for personal hygiene assistance.

In this study, most of the nurses (59.70%) had an average support for PCC, while 38.70% had a high support for PCC.

This study found no significant associations between the sociodemographic variables of gender, age, years of experience, and highest qualification and the total scores of the ICS-A-Nurse. Alhalal, Alrashidi and Alanazi (2020) found a positive association between the age of nurses and PCC provision, concluding that older nurses have greater abilities in PCC practice compared to younger nurses. Malfait, Eeckloo and Van Hecke (2017) attributed this finding to the effectiveness of older nurses in shared decision-making practices and their ability to involve patients actively in their care. Also, contrasting to the results from this study, Abu Lebda, Malak and Hamaideh (2020) also highlighted a relationship between gender and PCC, in which males had higher PCC compared to females. The males are more courageous than females in attempting to engage in any serious cases during patient care (Abu Lebda et al. 2020). Females have a high professional commitment to avoiding problems, so they may close any pathway that leads to accountability resulting from being involved in patient care (eds. Bulman & Schutz 2013). In Chang et al.’s (2020) study, there was a positive association between sociodemographic variables and the provision of PCC and experienced nurses were more committed to the practice of PCC than nurses with less experience.

### Limitations

The study was conducted with a small sample size in a specific research setting, which limits the generalisability of the findings to other contexts in South Africa. Furthermore, using a self-reported questionnaire to assess PCC from the nurses’ perspectives generated bias into the study setting (Alhalal et al. 2020). Future research should focus on patients’ perspectives when measuring PCC. However, this study took place during a global pandemic that significantly affected CCUs. Therefore, sharing information about PCC during a health pandemic, especially in a place with few resources, has important clinical and policy implications for making interventions that are tailored to the situation and aim to improve quality, patient-centred healthcare outcomes. In this study, only the perspectives of critical care nurses were evaluated. Further research into the perspective of the multidisciplinary team on PCC as well as the patients themselves is also required and may prove beneficial. Extending the research setting to include other medical facilities may be useful. A qualitative approach to the research that seeks to uncover individual experiences and interactions is desirable.

## Conclusion

Critical care nurses showed an average support for PCC. Therefore, critical care training and education programmes should include a PCC-focused approach. Ongoing in-service training to supplement existing knowledge on PCC should be implemented. To strengthen a PCC approach in clinical practice, the continual implementation of PCC initiatives should be a priority.
